# Application of Machine Learning Methods in Nursing Home Research

**DOI:** 10.3390/ijerph17176234

**Published:** 2020-08-27

**Authors:** Soo-Kyoung Lee, Jinhyun Ahn, Juh Hyun Shin, Ji Yeon Lee

**Affiliations:** 1 College of Nursing, Keimyung University, 1095, Dalgubeol-daero, Dalseo-gu, Daegu 42601, Korea; soo1005@kmu.ac.kr; 2 Department of Management Information Systems, Jeju National University, Jeju-do 63243, Korea; jha@jejunu.ac.kr; 3 College of Nursing, Ewha Womans University, Seoul 03760, Korea; dlwldusking@hanmail.net

**Keywords:** machine learning, accidental falls, nursing homes

## Abstract

*Background:* A machine learning (ML) system is able to construct algorithms to continue improving predictions and generate automated knowledge through data-driven predictors or decisions. Objective: The purpose of this study was to compare six ML methods (random forest (RF), logistics regression, linear support vector machine (SVM), polynomial SVM, radial SVM, and sigmoid SVM) of predicting falls in nursing homes (NHs). *Methods:* We applied three representative six-ML algorithms to the preprocessed dataset to develop a prediction model (*N* = 60). We used an accuracy measure to evaluate prediction models. *Results:* RF was the most accurate model (0.883), followed by the logistic regression model, SVM linear, and polynomial SVM (0.867). Conclusions: RF was a powerful algorithm to discern predictors of falls in NHs. For effective fall management, researchers should consider organizational characteristics as well as personal factors. *Recommendations for Future Research:* To confirm the superiority of ML in NH research, future studies are required to discern additional potential factors using newly introduced ML methods.

## 1. Background

The most frequently reported adverse event among nursing home (NH) residents, falls are associated with increased morbidity and mortality, and with reduced functioning [1,2,3]. The U.S. fall prevalence is 1.7 falls per resident yearly in NHs [1]; about 50% of NH residents in developed countries fall each year [4]. Accurate prediction of the factors associated with falls in NHs is important because nurses, health care professionals, researchers in practice, administrative staff, researchers, and politicians address fall issues. These stakeholders develop targeted fall-prevention management, and assess residents based on factors associated with a fall [5]. Prior studies identified a number of risk factors for falls; including age, sex, visual deficits, psychotropic medications, cognitive dysfunction [6,7,8], range of motion, urinary incontinence [9,10,11], hours per resident day (HPRD) of registered nurses (RNs), and skill mix [12].

Many prior studies on factors predicting falls in NHs have mainly used traditional regression analysis data [13,14]. However, traditional regression analysis has limitations in addressing the variability in data, nonlinear interactions of variables, and diverse distributions of datasets, which usually require basic assumptions [15]. To offset these limitations, researchers recently introduced a new approach: machine learning (ML). ML is a new computer-science study area using statistics, optimization methods, or artificial intelligence to gain statistical algorithms retrieved from a real dataset [15]. With input data, ML is able to construct algorithms that can continue improving predictions and generate automated knowledge through data-driven predictors or decisions. ML is especially beneficial when researchers work to discern relevant dependent features [16]. ML is a valid substitute for traditional statistical analysis in predicting conditional probabilities [17]. Using ML, researchers may report latent variables that were not usually reported using traditional data-analysis methods. ML also helps in providing clinical-decision support [15]. Because of these advantages, ML methods were recently introduced in the fields of health, medicine, and nursing to predict indicators such as mortality rates after radical cystesctomy [15], mortality-risk predictors for patients with burn injuries [17], health-related quality of life for elders with chronic disease [18], and NH residents’ visits to the emergency department as a predictor. In these studies, ML successfully identified mortality predictors, emergency department visits, and quality-of-life-related factors [15,18,19]. The dataset used in NH research and practice is becoming more dimensional, complex, and abundant, with advanced informatics and data management [20]. Furthermore, factors identified as fall-related factors in NHs are not independent of each other [21,22]. Considering the characteristics of these NH datasets, it is necessary to introduce ML when analyzing data from NHs.

One form of ML, the random forest (RF) algorithm, is applicable when researchers have more predictors than observations, based on the theory of ensemble learning that allows an algorithm to learn simple and complex classification functions accurately. RF does not require fine-tuning of parameters. Default parameterization often leads to excellent performance [23]. The support-vector-machine (SVM) model looks for a super-plane set of a high- or infinite-level space and uses it to perform classification and regression. SVM can learn complex classification functions efficiently, and employs powerful regularization principles to avoid overfitting [24]. One SVM, SVM linear, is a single parameter classification function where the parameter biases the test along the regression output values of the SVM. Researchers use SVM linear to handle large amounts of data vectors, such as text categorization. Moreover, researchers often use the SVM polynomial to process images, and the SVM radial when they have no prior information about the data. Mainly, researchers use SVM sigmoid as a proxy for neural networks [25].

The appropriate choice of the prediction model is challenging for nursing researchers. In this study, we introduced possible data analysis or interpretation in the field of nursing. The purpose of this study was to compare six ML methods (RF, logistics regression, linear SVM, polynomial SVM, radial SVM, and sigmoid SVM) in six aspects (accuracy, sensitivity, specificity, positive predictor values, negative predictor values, and receiver-operating characteristic curve) by demonstrating real data-based predicted factors related to resident falls. Furthermore, we aimed to identify predicted factors related to falls in NHs according to ML methods. Our prediction of fall risks enables nurses in NHs to support clinical discussions of risk factors and prevention strategies to lower fall incidence.

## 2. Methods

### 2.1. Study Design

This study used an NH dataset to compare various ML methods by demonstrating real data-based predicted factors related to resident falls.

### 2.2. Ethical Considerations

We obtained approval for this study from a university in Korea (IRB #136-4). We protected the confidentiality of participating organizations.

### 2.3. Measurements/Instruments

#### 2.3.1. Prediction Variables

Information on resident capacity, number of current residents, organization location, ownership, religious affiliation, affiliated hospitals, residents’ age, residents’ long-term-care insurance grade, residents’ sex, and long-term-care facility grade came from participating organizations. Administrators and directors of nursing (DONs) at each participating NH offered staff-related data. HPRD referenced mean hours worked by each type of staff (RNs, certified nurse aids, care workers, DONs, secretary general, social worker, dietician, and administrative staff) divided by the total number of residents [26]. The operational definition of skill mix was the proportion of RNs among the nursing staff and care workers. To calculate staff turnover, we calculated the total quantity of present staff at the end of the calendar year, the quantity of terminations in a calendar year, and turnover per year using the Nursing Facility Staff Survey.

#### 2.3.2. Outcomes Variable

We selected the variable *falls* as the outcome variable. We collected and distinguished between a residents’ group having no falls in the past 3 months (classified as 0) and another group having at least one fall in the past 3 months (classified as 1). Administrators, DONs, or nurses at each participating NH also provided fall-related data based on chart reviews.

### 2.4. Data Collection/Procedure

We retrieved the dataset employed in this study from a 3-year research project (2017–2020), Estimating Optimal Nurse Staffing for Nursing Home Residents Using an Optimization Model, funded by the Korea National Research Foundation. The principal investigator of this paper conducted the parent study on optimized nurse staffing HPRD for the best quality-care outcomes. In the parent study, from 2017 to 2020, the principal investigator or researchers visited NHs across South Korea from a random sample that agreed to participate, introducing the parent study to administrators or DONs and obtaining informed-consent forms. Administrators, DONs, or nurses filled out the survey by reviewing residents’ charts, nurses’ notes, and accident reports at the organizational level. From 2017 to 2020, researchers collected the data every 4 months. For this analysis, we retrieved only the first measured dataset: 60 NHs in 17 provinces of South Korea, that completed the survey from May 2017 to August 2017.

### 2.5. Data Analysis

#### 2.5.1. Statistical Data Analysis

We used SPSS version 23.0 (IBM Corporation, Armonk, NY, USA) for descriptive statistical tests for participating NHs’ characteristics.

#### 2.5.2. Machine Learning

**Figure 1:**
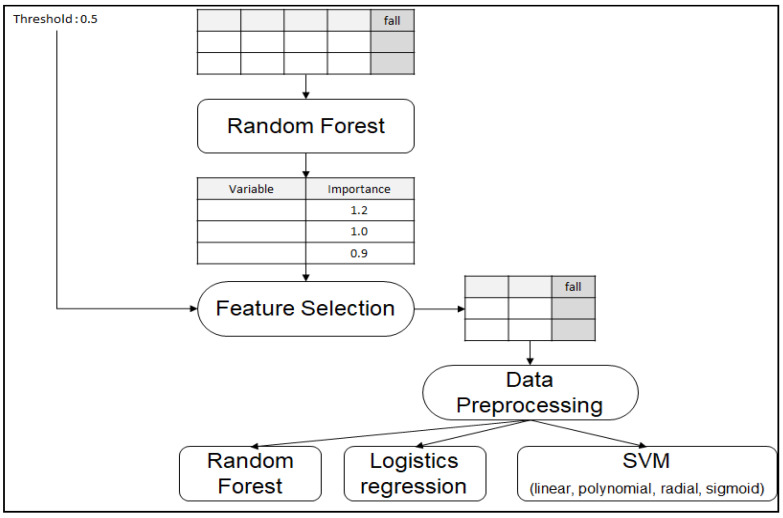
Process of data analysis.

Figure 1 depicts the two steps of workflow of this study: feature selection and data preprocessing. Because the collected dataset has 57 independent variables, it was infeasible to try every combination of variables. Selected features from RF follow (see Table 1). Table 1 shows the association of 10 variables, identified by ML, with falls. Using RF procedures, we identified six variables as predictors of falls, including HPRD of administrative staff; number of NH residents with psychiatric medications, aggressive behaviors, or cognitive dysfunction; urinary incontinence; current number of residents in each NH; and the maximum capacity of each NH. Logistic regression, polynomial SVM, and radial SVM identified only four predictors, whereas linear SVM and sigmoid SVM identified five predictors. We used RF to single out nonsignificant variables, reducing the number of independent variables to 13. The data-preprocessing step normalized values to supply the dataset to ML algorithms. We applied three representative ML algorithms—RF, logistics regression, and SVM (linear, polynomial, radial, sigmoid)—to the preprocessed dataset from a prediction model. We used ML algorithms in R programming (v3.3.1) to develop a prediction model.

### 2.6. Variable Selection

**Figure 2:**
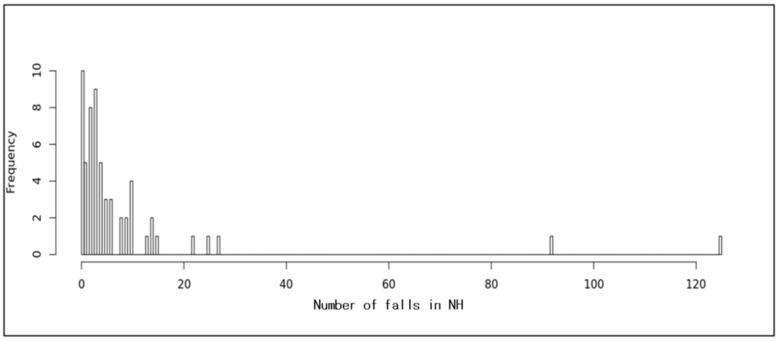
Histogram of falls.

We selected the variable falls as the dependent variable. Figure 2 indicates the frequency of falls in NHs. The histogram refers to the distribution of the number of falls in each nursing home. The minimum number of falls is 0 with a maximum value of 120, and the highest relative frequency (%) of residents who fall in each nursing home is less than 10%. In our prediction task, we distinguished between a residents’ group having no falls (classified as 0) and another group having at least one fall (classified as 1). We had 57 independent variables. Because the combination of independent variables was 144,115,188,075,855,870, it was infeasible to create a model to evaluate every combination. Thus, we used RF to select significant variables, allowing us to draw importance scores for each variable. Table 2 shows a list of significant variables ordered by importance scores that are greater than 0.5. We chose the threshold score of 0.5 because it was the most feasible combination based on a consultation with a statistics expert, as there are no scientific criteria for the threshold score.

### 2.7. Data Preprocessing

In the data-preprocessing step, we discarded meaningless variables, encoded them into integer numbers, and normalized these values. We discarded variables irrelevant to the prediction of falls in NHs: institution identification, name, evaluation time (the date of evaluation performance), year of institution establishment, and province. For categorical variables, we normalized values into integers to reflect grades. For example, facility-grade variables had values such as A, B, C, D, new, grading exception, and evaluation nontarget; we assigned integers 4, 3, 2, 1, 0, 0, and 0, respectively. We assumed Grade A was the best score and D the worst. We assigned values of zero when no evaluation was performed. Religious values had already been encoded into integers except for string values, such as “5 (Won Buddhism)”; it was trivial to convert the string to 5. Some variables represented values in ratio form. The variable Skill_mix_RN_CNA represents the ratio of the number of nurses to the number of nursing assistants (CNAs). For example, the value 1:4 means the number of CNAs is four times the number of nurses. We converted the value to 0.25.

### 2.8. Predicting Modeling

We applied three ML algorithms to the preprocessed dataset. We measured the baseline by applying the RF method first to optimize the matrix of the prediction model. RF is an ensemble ML method that constructs multiple decision trees at training time [27]. The output class rests on the mean prediction of individual decision trees. RF overcomes the overfitting problem of conventional decision-tree methods, generating multiple decision trees at training time.

Researchers widely use logistic regression; the ML method uses a logistic function to address two class-dependent variables [28]. SVM, also a widely used ML method, tries to find a support vector by correctly dividing given training data in a featured space, constructed by a kernel function [29].

We used R packages to run these ML algorithms. For RF, we set the parameter ntree to 100, representing the number of generated trees. We observed no significant improvement when the value of ntree was bigger. For logistic regression and SVM, we used default parameter-value settings. We used four well-known kernel functions in SVM: linear, polynomial, radial, and sigmoid. Based on this configuration, we had six models.

### 2.9. Evaluation of Prediction Models

To validate each prediction model, we used five-fold cross validation, dividing the data set into five parts, then training in four and testing in one, repeating the process until testing all parts. We built a confusion matrix to gauge each model’s performance, measured with six parameters: accuracy, sensitivity, specificity, positive predictor values, negative predictor values, and receiver-operating characteristic curve. We assigned the accuracy measure the highest importance because the main goal of the study was to find a prediction model that best predicted the variables [30].

## 3. Results

### 3.1. Characteristics of the Organizations (n = 60)

Table 3 summarizes descriptive statistics of participating NHs. Of NHs, 70% were located in metropolitan or urban areas (population over half million); about 82% were not for profit, about 35.0% affiliated with a religion, and 90% affiliated with a hospital. Residents’ average age was 83.6 (*SD* = 2.404). About 34.4% of residents had first and second ratings, most of whom were bedridden with severe activities of daily living limitations. Of NHs, 38.3% received a superior grade (Grade A) in the long-term care facility evaluation. The average number of nurses was 1.48, the HPRD for nurses was 0.191 (11 min and 30 s), and the turnover rate was 9.497%.

### 3.2. Predicting Modeling

We used R packages to run these ML algorithms. For RF, we set the parameter ntree to 100, which represented the number of generated trees. We observed no significant improvement when the value of ntree was bigger. For logistic regression and SVM, we used default parameter-value settings. We used four well-known kernel functions in SVM: linear, polynomial, radial, and sigmoid. We had six models based on this configuration.

**Figure 3:**
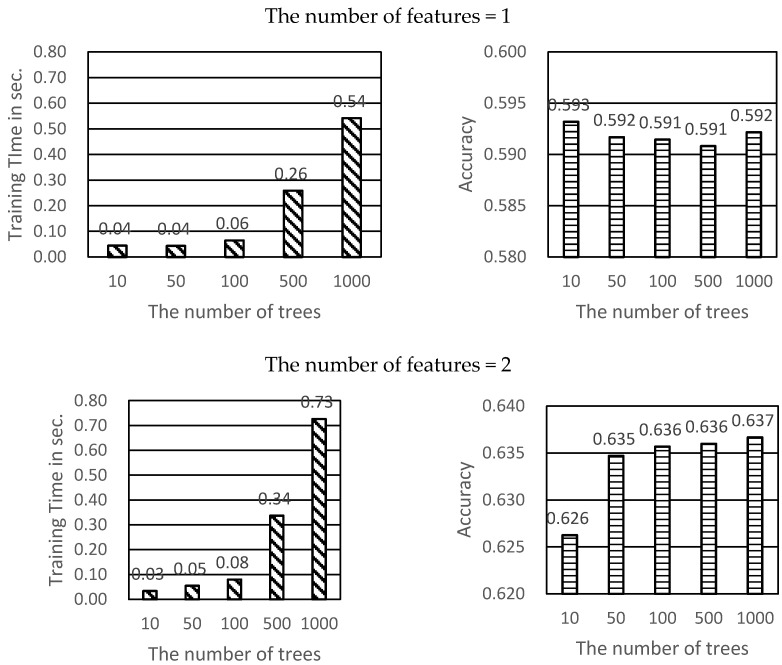
Predicting Modeling by default parameter value.

To perform tuning on the most important parameter of ntree, the accuracy and training time was examined by calculating 10, 50, 500, 1000 ntree combined by the feature 1 or 2 respectively (see Figure 3). The average training time of Feature 1 was 0.03 s for 10 ntree and 0.06 s for 100 ntree. The average training time dramatically increased to 0.26 s for 500 ntree. However, there was no difference in terms of ntree accuracy. For the two features, the accuracy was increased because the value of the ntree increased slightly. The accuracy was 0.636 for 100 ntree and 0.636 for 1000 ntree. However, the training time dramatically increased from 0.08 s for 100 ntree to 0.34 s for 500 ntree. Thus, it can be said that the ntree of 100 was most accurate for parameters with short training time.

### 3.3. Predictive Performance

**Figure 4:**
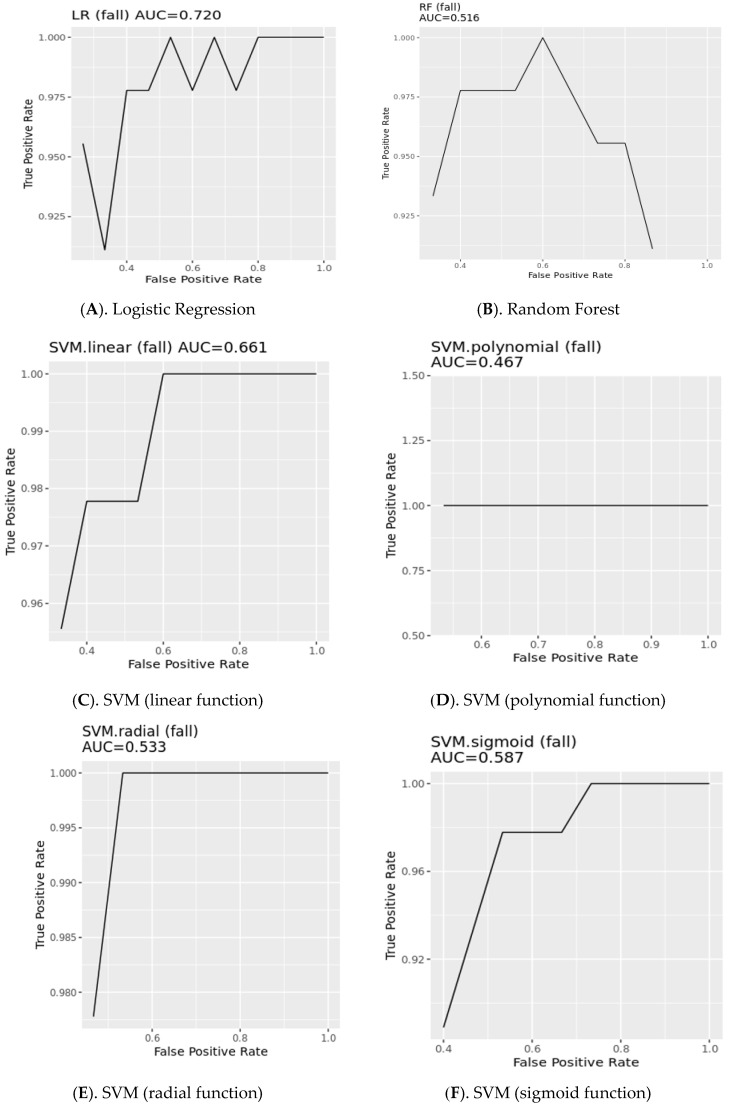
Area under the curve of prediction models.

**Figure 5:**
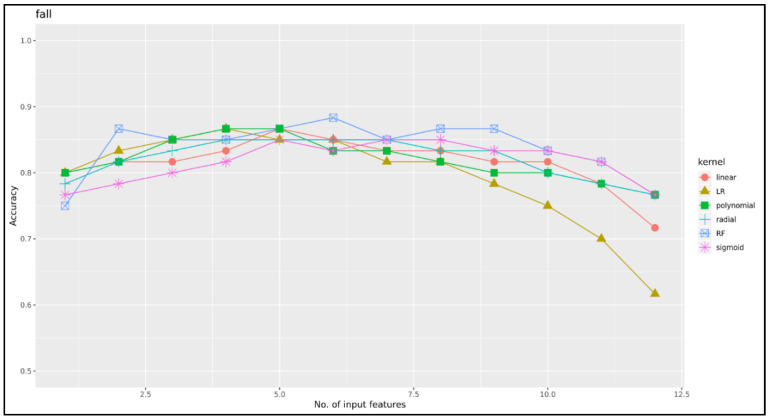
Kernel function of support vector machine.

Table 4 shows performance and Figure 4 shows the corresponding area under the curve: the RF model had the greatest accuracy (0.883). After the logistic regression model (0.867), the linear and polynomial SVM (0.867), and radial and sigmoid SVM (0.850) models had decreasing orders of performance. The reason for the reduced receiver operating characteristic shown in Figure 4B is that RF uses a combination of multiple decision trees to categorize them, so the overall accuracy rate can be very poor because some decision trees are often overfitted. Figure 5 depicts the comparison of the accuracy of the ML algorithms: logistics regression, RF, and SVM (linear, polynomial, radial, sigmoid). Figure 5 shows that using many features does not improve performance. The best accuracy of the logistic regression model was 0.867 and included four variables as predictors of falls, including CNA_HPRD, hours per resident day of the secretary general, proportion of residents with urinary incontinence, and proportion of residents with psychiatric medications. The best accuracy of the Random Forest Model was 0.883 and included six variables as predictors of falls, including proportion of residents with psychiatric medications, proportion of residents with urinary incontinence, current number of residents in a nursing home, proportion of residents with aggressive behavior, proportion of residents with cognitive decline, and maximum capacity of residents. The best accuracy of the SVM linear model was 0.867 and included five variables as predictors of falls, including hours per resident day of care workers, current number of residents in a nursing home, CNA_HPRD, turnover of care workers, and hours per resident day of the secretary general. The best accuracy of the SVM polynomial model was 0.867 and included four variables as predictors of falls, including current number of residents in a nursing home, hours per resident day of the secretary general, proportion of residents with urinary incontinence, and proportion of residents with psychiatric medications. The best accuracy of the SVM radial model was 0.850 and included four variables as predictors of falls, including hours per resident day of care workers, hours per resident day of the secretary general, proportion of residents with cognitive decline, and proportion of residents with psychiatric medications. The best accuracy of the SVM sigmoid model was 0.850 and included five variables as predictors of falls, including hours per resident day of care workers, CNA_HPRD, hours per resident day of the secretary general, proportion of residents with aggressive behavior, and proportion of residents with urinary incontinence.

Logistic regression, SVM polynomial, and SVM radial identified only four predictors, whereas SVM linear and sigmoid identified five predictors. The strongest predicting variable was HPRD of the secretary general, supported by logistic regression, linear SVM, polynomial SVM, radial SVM, and sigmoid SVM. The proportion of residents with psychiatric medications was supported by logistic regression, RF, polynomial SVM, radial SVM, and sigmoid SVM. The proportion of residents with urinary incontinence was supported by logistics regression, RF, polynomial SVM, radial SVM, and sigmoid SVM.

## 4. Discussion

RF reported more accurate values than other algorithms including linear/polynomial/radial/sigmoid SVM and regression in this study. Previous studies reported the accuracy of memetic algorithms; a meta-analysis reported that SVM was more accurate than an artificial neural network decision tree, naïve Bayes, and K-nearest neighbor [31,32,33]. RF reported the same value as sigmoid SVM, but has greater specificity values than other algorithms including linear/polynomial/radial SVM and regression in this study. This means the RF has ability to predict true negatives of each available category [31]. The RF reported more positive predictive values than other algorithms including linear/polynomial/radial/sigmoid SVM and regression in this study. The RF is useful in predicting falls due to its higher positive predictive value [32]. Classical statistical methods may report only similar results because of difficulties in addressing big data. However, ML was designed to analyze raw datasets without specific interpretation or refinement of data, and can report related factors that were not usually reported in previous research. This study applied many ML methods to discern the best-fit method.

The proportion of residents prescribed psychiatric medications and urinary incontinence were top factors in the logistic regression, RF, polynomial SVM, radial SVM, and sigmoid SVM. Psychiatric medication was a very strong predictor of falls, consistent with previous research [6,7,9,10], but inconsistent with a couple of studies [34,35]. The proportion of residents with urinary incontinence was an important factor in falls, supported in previous research [9,10,11,12], but not supported in other studies [7,32]. Results confirmed that the frequency of resident falls relates to increased psychiatric medication and urinary incontinence [9,12,36,37]. No practical guide exists for NH staff based on scientific evidence [38]. Related factors supported in this study should be included in guidelines: careful medication administration by professional RNs and a healthy environment would reduce falls, considering the case mix of residents [1,39].

Five ML algorithms predicted the HPRD of the secretary general, inconsistent with previous research [10]. The logistic regression, linear SVM, and sigmoid SVM also supported the HPRD of CNAs as a predictor of falls, supported in previous research [40,41,42] but inconsistent with other research [43,44]. The number of care workers was another important factor for falls, supported in linear SVM, radial SVM, and sigmoid SVM, but not supported in Shin [43] and Shin and Hyun [44]. Remarkably, administrative staff was an important factor in resident falls using ML analysis. Very limited studies [10] report that the stability of administrative staff is related to resident falls. A scientific and sound interdisciplinary approach among NH staff requires teamwork among nurses and administrative staff, including shared governance [45,46,47].

Only RF reported that the total number of residents was an important factor in falls, consistent with Thomas et al. [42], but not with Van Doorn et al. [7]. The RF and SVM radical reported that the proportion of residents with cognitive dysfunction related to falls, consistent with preceding research [6,7,8], but inconsistent with Datta et al. [34].

This study has some limitations. First, although we provided the definition of falls to data collectors in each NH, they may have underestimated or overestimated the number of falls; we could collect only self-reported data from each NH. Moreover, we did not collect the specific location or date of falls; rather, we asked how many slips or falls took place in the last quarter. Furthermore, we did not differentiate between types of falls, slips, and fall-related injuries in this study; future studies should consider these factors. Second, the sample size is too small to train a stable and satisfied ML model considering the number of original features. However, we tried to offset this limitation by querying 60 NHs across Korea. Clearly, more research is required with a large sample size to confirm relationships. Third, parameter tuning, which is important for increasing model performance, was missed in this study. Future research is required using parameter tuning.

## 5. Conclusions

This study applied six ML methods to predict factors related to falls of NH residents. We validated the scientific data analysis by developing six ML algorithms, applied to a dataset from 60 NHs across Korea. The RF model had the greatest accuracy (0.883), after the logistic regression model (0.867), the linear and polynomial SVM (0.867), radial and sigmoid SVM (0.850) models had decreasing orders of performance in predicting falls. We propose RF as a powerful algorithm to discern predictors of falls in NHs.

We also identified many predicted factors related to falls in NHs according to ML methods, including individual factors (proportion of residents with psychiatric medication, urinary incontinence, aggressive behavior or cognitive decline) and organizational factors (current number of residents in the NH and maximum capacity of residents). We concluded that both organizational and personal factors should be considered for effective fall management for NH residents. It was confirmed that the variable is a factor in predicting the fall.

### Recommendations for Future Research

Examination of related organizational factors on falls is quite meaningful, in that preventing falls contributes to improving the quality of life, care of residents, and decreasing health care costs. Future research is needed to demonstrate different and diverse ML algorithms based on the large dataset of nursing home data to confirm its potential factors.

## Figures and Tables

**Table 1:** Predicting Variables of Six Models.

Model	Hours Per Resident Day of Secretary General	Proportion of Residents with Psychiatric Medication	Proportion of Residents with Urinary Incontinence	Hours Per Resident Day of Care Worker	Current Number of Resident in a Nursing Home	CNA_HPRD	Proportion of Residents with Aggressive Behavior	Proportion of Residents with Cognitive Decline	Turnover of Care Worker	Maximum Capacity of Residents	Total
Logistic regression	O	O	O			O					4
Random forest		O	O		O		O	O		O	6
SVM linear	O			O	O	O			O		5
SVM polynomial	O	O	O		O						4
SVM radial	O	O		O				O			4
SVM sigmoid	O		O	O		O	O				5
-	5	4	4	3	3	3	2	2	1	1	-

*Note.* CNA = certified nurse aide, HPRD = hours per resident day, SVM = support vector machine.

**Table 2:** Description of variables.

No.	Variable	Importance Score
1	Hours per resident day of the secretary general	1.8334
2	Proportion of residents with psychiatric medications	1.5208
3	Proportion of residents with urinary incontinence	1.2823
4	Hours per resident day of care workers	1.0291
5	Current number of residents in a nursing home	0.8903
6	CNA_HPRD	0.8418
7	Proportion of residents with aggressive behavior	0.7985
8	Proportion of residents with cognitive decline	0.7785
9	Turnover of care worker	0.7754
10	Maximum capacity of residents	0.6628

*Note.* CNA = certified nurse aide, HPRD = hours per resident day.

**Table 3:** Characteristics of Nursing Homes (*n* = 60).

Variable	Frequency	%	*M*	*SD*	Min	Max
Capacity			75.13	55.83	7	296
Number of current residents			66.73	48.51	7	295
Location of organizations						
Metropolitan (over million)	29	48.3				
Urban location (over round half million)	13	21.7				
Local small city (5–50 thousand)	13	21.7				
Rural area (less than 50 thousand)	5	8.3				
Ownership						
Profit	10	16.7				
Not for profit	49	81.7				
Religious affiliation						
No religion	39	65.0				
Christianity	11	18.5				
Catholic	3	5.5				
Buddhism	3	5.5				
Others	3	5.5				
Affiliated hospitals						
No	5	8.3				
Yes	54	90.0				
Average age of residents			83.60	2.404	80	89
Determined Long-term care insurance grade by the Korean National Health Insurance Corporation (%)						
1st ^a^			12.00	8.82	0	41
2nd ^b^			22.40	11.46	0	60
3rd ^c^			35.95	14.39	0	90
4th ^d^			22.41	16.95	0	90
5th ^e^			1.18	3.00	0	15
Unrated			5.98	19.48	0	100
Gender (%)						
Male			20.77	11.35	0	64
Female			78.95	11.30	36	100
Long-term care facility grade (%)						
Grade A (Superior)	23	38.3				
Grade B (Above average)	8	13.3				
Grade C (Average)	6	10.0				
Grade D (Below average)	7	11.7				
Grade E (Poor)	0	0.0				
Ungraded	16	26.7				
Number of registered nurses			1.48	2.89	0	17
Number of certified nurse aides			1.95	1.44	0	6
Number of care worker			26.58	20.52	1	132
Hours per resident day						
Registered nurses			0.19	0.24	0.00	1.03
Certified nurse aides			0.36	0.26	0.00	1.42
Care worker			3.82	1.63	1.32	9.76
Director			0.26	0.23	0.03	1.42
Secretary general			0.12	0.12	0.00	0.51
Social worker			0.39	0.27	0.00	1.42
Dietician			0.09	0.09	0.00	0.36
Administrative staff			0.14	0.22	0.00	1.36
Turnover (%)						
Registered nurses			9.49	21.61	0.00	100.00
Certified nurse aides			12.54	22.49	0.00	66.66
Care worker			19.06	18.50	0.00	86.17
Director			4.17	19.07	0.00	20.15
Secretary general			12.23	33.12	0.00	83.17
Social worker			17.99	29.49	0.00	81.22
Dietician			29.83	42.65	0.00	91.15
Cook			38.46	36.31	0.00	95.27
Administrative staff			21.39	35.17	0.00	88.19

Note. ^a^ Nursing Home Residents who are completely dependent for activities of daily living. ^b^ Nursing Home residents who are mostly dependent for activities of daily living. ^c^ Nursing Home Residents who are partially dependent for activities of daily living. ^d^ Nursing Home residents who have limited dependence for activities of daily living. ^e^ Nursing Home residents with dementia.

**Table 4:** Comparison of Performance in Prediction Models.

Model	Accuracy	Sensitivity	Specificity	PPV	NPV
Logistic regression	0.867	1.000	0.467	0.849	1.000
Random forest	0.883	0.978	0.600	0.880	0.900
SVM linear	0.867	0.978	0.533	0.863	0.889
SVM polynomial	0.867	1.000	0.467	0.849	1.000
SVM radial	0.850	0.978	0.467	0.846	0.875
SVM sigmoid	0.850	0.933	0.600	0.875	0.750

Note. PPV = positive predictive values, NPV = negative predictive values, SVM = support vector machine.

## Data Availability

The datasets generated and analyzed during the current study are not publicly available because the agreement indicated they be used only for the purpose of this study when participants’ consent was obtained.

## Abbreviations

CNA	Certified nursing assistant
DON	Director of nursing
HPRD	Hours per resident day
ML	Machine learning
NH	Nursing Home
RF	Random forest
RN	Registered nurse
SVM	Support vector machine
